# A High-Precision Bandgap Reference with Chopper Stabilization and V-Curve Compensation Technique

**DOI:** 10.3390/mi15010074

**Published:** 2023-12-29

**Authors:** Enming Chen, Thomas Wu, Jianhai Yu, Liang Yin

**Affiliations:** 1 School of Computer, Electronics and Information, Guangxi University, Nanning 530004, China; 2113301004@st.gxu.edu.cn (E.C.); xwu@gxu.edu.cn (T.W.); 2 The Guangxi Key Laboratory of Machine Vision and Intelligent Control, Wuzhou University, Wuzhou 543002, China; 3 MEMS Center, Harbin Institute of Technology, Harbin 150001, China; yinliang2003@126.com

**Keywords:** bandgap reference, chopper stabilization, V-curve compensation, MEMS sensor interface circuit

## Abstract

The MEMS sensor converts the physical signal of nature into an electrical signal. The output signal of the MEMS sensor is so weak and basically in the low-frequency band that the MEMS sensor interface circuit has a rigorous requirement for the noise/offset and temperature coefficient, especially in the bandgap reference block. However, the traditional amplifier has low-frequency noise and offset voltage, which will decrease the precision of the bandgap reference. In order to satisfy the need of the MEMS sensor interface circuit, a high-precision and low-noise bandgap reference is proposed in this paper. A novel operational amplifier with a chopper-stabilization technique is adopted to reduce offset and low-frequency noise. At the same time, the V-curve compensation circuit is used to realize the second-order curvature compensation. The circuit is implemented under the 0.18 μm standard of the CMOS process. The test result shows that the temperature coefficient of the bandgap is 2.31 ppm/°C in the range of −40–140 °C, while the output voltage noise is only 616 nV/sqrt(Hz)@1 Hz and the power-supply rejection ratio is 73 dB@10 kHz. The linear adjustment rate is 0.33 mV/V for supply voltages of 1.2–1.8 V at room temperature, the power consumption is only 107 μW at 1.8 V power supply voltage, and the chip active area is 0.21 × 0.28 mm^2^.

## 1. Introduction

With the rapid advancement in microelectronics, MEMS (Micro-Electro–Mechanical Systems) sensors, which feature small size, an ability to work in an extreme environment, and a high signal-to-noise ratio, have found widespread applications in diverse fields, such as geophysical exploration, aerospace technology, IoTs(Internet of Things) [1], and healthcare. MEMS technology integrates microelectronic devices with micromechanical components on a single silicon chip, which involves a variety of disciplines and technologies. The characteristic dimensions of MEMS devices can be in the micrometer scale, allowing for mass production and manufacturing processes similar to those used in integrated circuits. Moreover, MEMS devices can be seamlessly integrated with CMOS (Complementary Metal–Oxide–Semiconductor) processes, making them a focal point of research internationally. Despite significant challenges, substantial progress has been made and numerous cost-effective MEMS products have emerged. Due to their compact size, light weight, and ease of integration, MEMS devices are extensively used as control units in portable devices. A critical component of MEMS sensors is the interface circuitry, which acts as a conduit for converting sensing signals to digital outputs. The performance of this interface circuit significantly influences the sensor’s overall metrics. It primarily comprises a front-end analog signal-detection circuit and a Σ–Δ (Sigma–Delta) modulator. The front-end circuit includes a bandgap reference and an instrumentation amplifier. The bandgap reference provides a stable baseline voltage for the sensor-interface circuit system, necessitating attributes such as low-temperature drift and minimal output noise. 

The bandgap reference is widely used in analog and mixed-signal electronic devices, such as A/D converters, D/A converters, linear regulators, phase-locked loops, and operational amplifiers for their high precision and temperature independence [2,3,4,5,6,7]. In high-performance application circuits, the noise performance of each module plays a decisive role in the overall system performance [8,9,10,11,12]. The bandgap reference provides a DC reference for each module of the system [11], so the performance of the bandgap reference directly affects the various performance of the sensor. To reduce the output noise of the bandgap reference in order to avoid the deterioration of other modules, many techniques are adopted. In reference [11], the bandgap reference with the dual output structure, which includes three low-pass filters and two operational amplifiers, are proposed to reduce the output noise. However, the structure is complex, and a large resistance or large capacitor is often used in order to filter out the noise with low frequency. On the one hand, the integration of passive devices needs to occupy a large chip area. On the other hand, the large resistance and capacitance will lead to a very large time constant, which means the bandgap reference will need a long transient response time. In reference [13], the noise is suppressed by adopting the Darlington structure BJT, but it occupies more area than the traditional bipolar transistor. This structure has strict requirements on device parameter settings and inter-device matching. In reference [14], a notch filter is used as the output filter to further reduce the output voltage ripple. However, the increased capacitance used in the notch filter further increased the chip area. In reference [15,16], the bandgap reference with a chopper-stabilization technique [17] is proposed, which can improve circuit performance. However, in reference [15], the pre-regulator, SC filter, and output buffer will significantly increase power consumption and area consumption. In reference [16], the chopper switches are only located at the input and output ends, which increases the output noise and limits the bandwidth. In reference [18], a low-noise bandgap reference is proposed, which uses a simple topology to achieve a low output noise. However, the TC is 13.07 ppm/°C, meaning it cannot meet the requirement of the high-precision applications. In the MEMS sensor interface circuit, the ultra-low TC (temperature coefficient) is still required. While the traditional bandgap reference mostly uses the first-order compensation technique, typically achieving a minimum of approximately 20 ppm/°C, MEMS sensor interface circuits demand even lower TC. To further reduce the non-linear term and consequently decrease TC while enhancing output accuracy, it becomes necessary to implement high-order curvature compensation. Many studies on the second-order compensation of the bandgap reference have been completed in recent years. In reference [19], a combination of two BJTs and two MOSFETs, biased in their strong-inversion regions, is employed to generate a higher-order compensation signal. While this structure can achieve a low TC of sub-1 ppm/°C, the use of numerous resistors introduces the need for external trimming, and the resistors themselves have temperature coefficients, which can have a significant impact on the precision of the produced chips. In reference [20], the bandgap reference with the segmented curvature temperature-compensation technique is proposed to achieve a low TC over a wide temperature range with low power consumption. However, in order to realize the precise compensation, this structure needs many comparators and logic gates and resistors, which will significantly increase power consumption, and response time, and it will introduce noise and interference. In reference [21], the high-order components of the CTAT (Complementary to Absolute Temperature) current are eliminated by adopting different CTAT current differences and then compensating with PTAT (Proportional to Absolute Temperature). This ultimately cancels the thermal nonlinearity of the emitter-base voltage. However, the structure increases noise and significantly increases the complexity of the circuit design as multiple amplifiers have pole issues that need special frequency compensation to ensure circuit stability. Although the above studies realize low TC, their structures are too complicated. Not only are the chip area, power consumption, and the difficulty of design increased, but additional noise is also generated. A novel bandgap reference is proposed in this study, which uses the chopper stabilization and V-curve compensation technique in order to meet the requirement of MEMS sensor-interface circuits.

## 2. Materials and Methods

### 2.1. Chopper-Stabilization Technique

**Figure 1:**
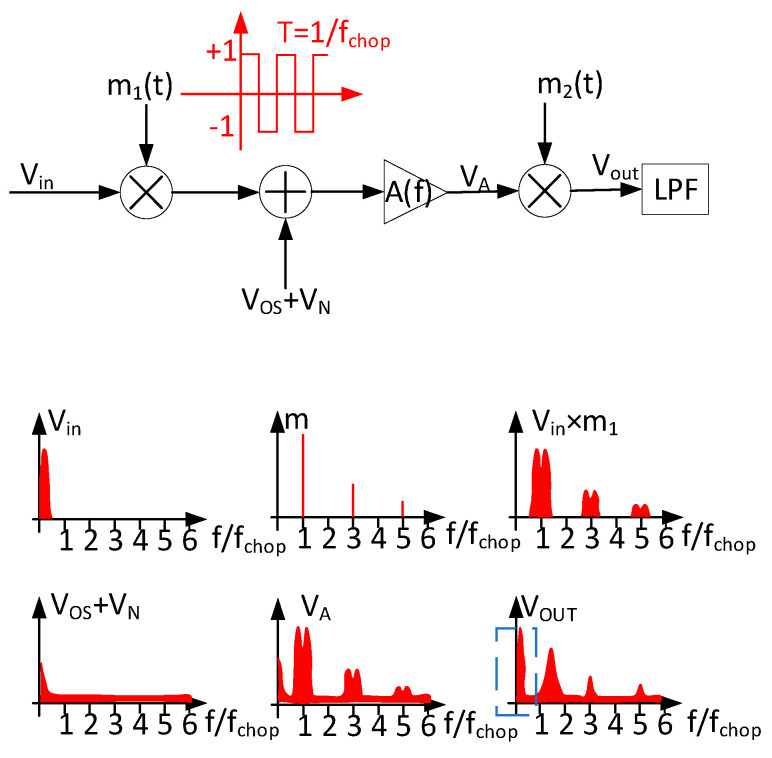
The chopper process and signal modulation and demodulation in frequency domain.

**Figure 2:**
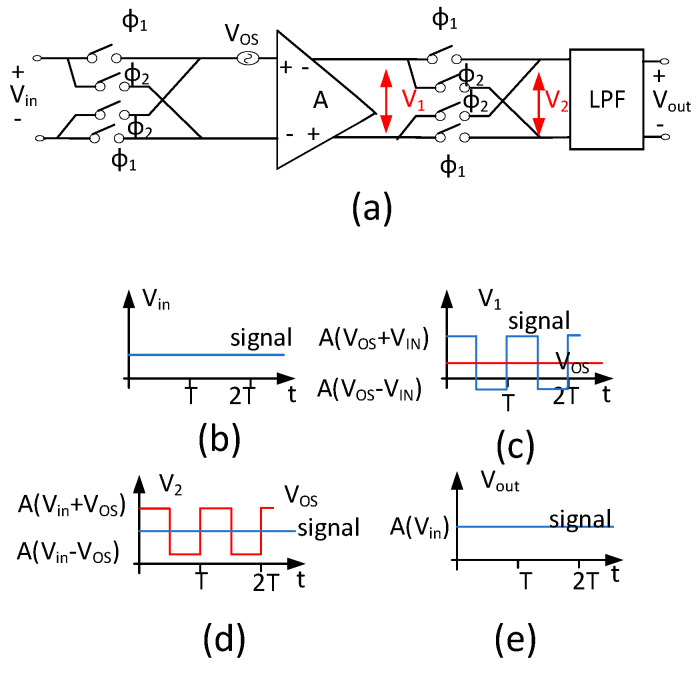
(**a**) The schematic of chopper; (**b**) Input signal; (**c**) The wave form of first modulation; (**d**) The wave form of second modulation; (**e**) Output signal.

In the MEMS sensor-interface circuit, there are many low-frequency noises and Vos (input offset voltage of amplifier), which will significantly impact its performance. The former originates primarily from the 1/f noise, while the latter results from random errors caused by transistor mismatch. In order to design a bandgap reference, which can be used in MEMS sensor-interface circuits, the chopper-stabilization technique is used in the proposed bandgap. The principle of the chopper modulation and demodulation technique is shown in Figure 1 and Figure 2 in the frequency and time domain, respectively. As shown in Figure 1, a square-wave signal m(t) with 50% duty cycle is used for modulation and demodulation. The Fourier transform expression of the square wave can be represented as Equation (1). According to this expression, the square wave only has components at the odd harmonic frequencies of f_chop_, which is the frequency of modulation signal m_1_(t) and m_2_(t). In the chopper-stabilization techniques, the chopper frequency is a critical parameter as it directly affects the performance of the entire chopper-operational amplifier. To amplify the signal from MEMS sensors, it is imperative that the chopper frequency be lower than the bandwidth of the amplifier. This is necessary to prevent significant gain loss in the amplifier. In addition to the amplifier’s bandwidth limitation, the aim of this study is to minimize 1/f noise as much as possible and ensure that the signal is not aliased after demodulation. In summary, this necessitates that the chopper frequency satisfies Equation (2) [22]. In this expression, f_corner_ represents the noise-corner frequency, while $B_{\omega ,signal}$ and f_c_ represent the signal bandwidth and the cutoff frequency of amplifier. If the f_chop_ exceeds f_c_, it will result in the amplifier filtering out a part of the modulated signal. Conversely, if f_chop_ is less than f_corner_+2$B_{\omega ,signal}$, it will lead to aliasing. The f_chop_ is generally chosen at about 1/5 times f_c_ [23] in order to preserve the integrity of the amplified signal and prevent aliasing. After the first modulation, the signal is modulated onto the odd harmonic components of f_chop_, which is at a high frequency. The modulated signal is then amplified by the amplifier, including the offset and low-frequency noise, resulting in the output signal V_A_. With the second modulation, the signal is demodulated back to the low-frequency band, while the offset and low-frequency noise are modulated to the high-frequency band. In the process of twice modulation, the signal is essentially multiplied by A × m^2^(t), which is equivalent to A × (16/$\pi^{2}$) × sin^2^(2$\pi f_{chop}$t) = A $\times (16/\pi^{2})\times \left(1-\cos\left(4\pi f_{chop}t\right)\right)/2$. The resulting output signal is V_OUT_, as shown in Figure 1, and the expression for the output voltage is provided by Equation (3). Finally, the pure amplified signal can be obtained through a low-pass filter. As shown in Figure 2c, the modulation is equivalent to the signal multiplied by +1 or −1, and then the V_OS_ and low-frequency noise are added to the modulated signal before passing into the amplifier. In the process of the second modulation, V_1_ is multiplied by +1 or −1, which is provided by A(V_OS_ + V_in_) ×1 or A(V_OS_ − V_in_) ×(−1). The wave form after the second modulation is V_2_, as shown in Figure 2d. In addition, the V_OS_ and low-frequency noise can be easily eliminated by removing the high-frequency components from the signal. As mentioned above, the amplitude of the DC component in the output signal is (8/π^2^)·AVin, and the gain of the chopper operational amplifier, decreases by approximately 20%. Hence, it is necessary to select an amplifier with a sufficiently high gain.

(1) $$m(t)=\overset{\infty }{\underset{k=1}{\sum }}\frac{4}{k\pi }\sin\left(\frac{k\pi }{2}\right)\sin\left({2\pi \mathrm{kf}}_{\mathrm{chop}}t\right)$$

(2) $$f_{\mathrm{corner}}+2B_{\omega ,\mathrm{signal}}<f_{\mathrm{chop}}<f_{c}$$

(3) $$V_{\mathrm{out}}(t)=A\times \left[V_{\mathrm{in}}\left(t\right)\times {m(t)+V}_{N}\left(t\right)\right]m(t)=A\times V_{\mathrm{in}}\left(t\right)\times {m(t)}^{2}+A\times V_{N}\left(t\right)\times m(t)$$

**Figure 3:**
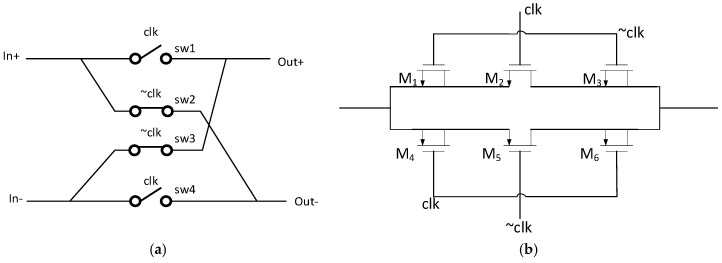
(**a**) Chopper structure; (**b**) The switch structure of chopper.

The schematic of the chopper is shown in Figure 3a, consisting of four clock-controlled switches. The clk and ~clk signals are non-overlapping, enabling the alternation of the positive and negative ends of the differential signals, which is equivalent to a multiplication of ±1. The structure of switch is depicted in Figure 3b. In this diagram, M_1_, M_3_, M_4_, and M_6_ represent virtual switches with an aspect ratio of 1/2 compared to M_2_ and M_5_. This structure can reduce charge injection and the impact of clock feedthrough. M_2_ and M_5_ are complementary switch structures in order to get a stable and low on-resistance. The on-resistance expression for this switch structure is provided by Equation (4). When the $\mu_{n}C_{ox}{\left(\frac{W}{L}\right)}_{2}=\mu_{p}C_{ox}{\left(\frac{W}{L}\right)}_{5}$, the impact of input level on the on-resistance can be minimized. Furthermore, as shown in the first term of the denominator in the Equation (4), increasing the aspect ratio of M_2_ can reduce the on-resistance. As mentioned above, M_5_ needs to be increased in accordance with the increase in M_2_. However, an excessively large aspect ratio can result in increased charge injection and introduce parasitic capacitance. Therefore, in consideration of minimizing the clock feedthrough effect, the chopper switch (CH_1_) at the input end uses a smaller-sized NOMS transistor (1 μm/0.18 μm), while the NOMS transistor for CH_2_ and CH_3_ are chosen with a larger size (2 μm/0.18 μm) to reduce the voltage drop caused by the on-resistance.

(4) $$\begin{array}{cc} R_{on}= & R_{on,2}∥R_{on,5}\\ = & \frac{1}{\mu_{n}C_{ox}{\left(\frac{W}{L}\right)}_{2}\left(V_{DD}-V_{in}-V_{THN}\right)}∥\frac{1}{\mu_{p}C_{ox}{\left(\frac{W}{L}\right)}_{5}\left(V_{in}-\left|V_{THP}\right|\right)}\\ = & \frac{1}{\mu_{n}C_{ox}{\left(\frac{W}{L}\right)}_{2}\left(V_{DD}-V_{THN}\right)-\left[\mu_{n}C_{ox}{\left(\frac{W}{L}\right)}_{2}-\mu_{p}C_{ox}{\left(\frac{W}{L}\right)}_{5}\right]V_{in}-\mu_{p}C_{ox}{\left(\frac{W}{L}\right)}_{5}\left|V_{THP}\right|} \end{array}$$

**Figure 4:**
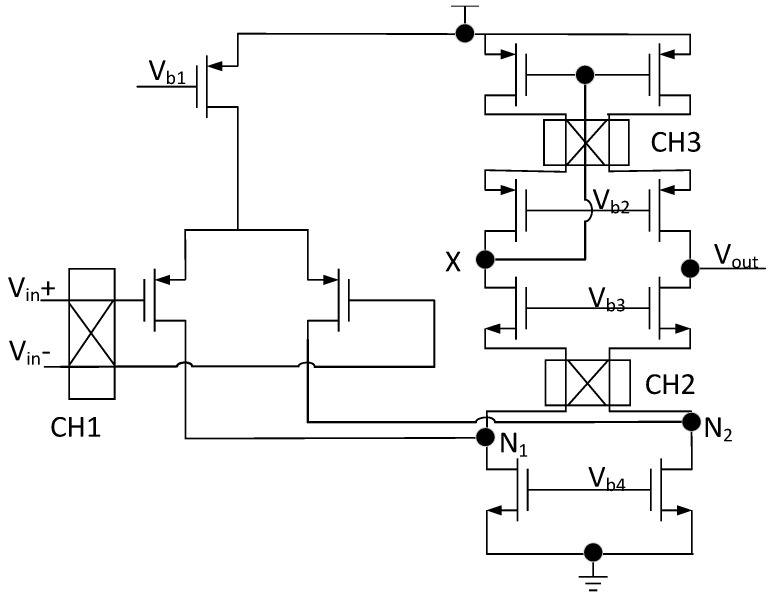
Chopper-operational amplifier structure.

In terms of the choice of chopper-operational amplifier, there are three commonly used operational-amplifier structures, including two-stage amplifier, cascode amplifier, and folded cascode amplifier. The two-stage amplifier has the largest output swing among these three structures, but it has low bandwidth, high power consumption, and low PSRR (Power-Supply Rejection Ratio). The cascode amplifier has a wide bandwidth, but its output swing is too small. Compared with above two structures, the folded cascode has a high bandwidth similar to the cascode amplifier. Furthermore, its output swing and power consumption fall between them. So, the folded cascode amplifier is suitable for our circuit. The structure of the chopper operational amplifier is shown in Figure 4,which is the folded cascode amplifier structure. The chopper-operational amplifier employs PMOS differential pairs as input transistors to reduce the impact of input noise on the operational amplifier. The pole formula ω = −1/RC suggests that when the output impedance is low, the pole’s value is relatively high. Placing a chopper switch at the low-impedance node can reduce the impact of parasitic capacitance of chopper on the amplifier bandwidth and not reduce the voltage swing amplitude. Therefore, modulator CH_2_ is placed at the folding point N_1_(N_2_), which is a low-output impedance point. To further reduce current mismatch in the current mirror and minimize input offset, the modulator CH_3_ is placed at the intermediate node of the low-voltage cascode current mirror. This additional chopper-operational amplifier is utilized for dynamically switching the two PMOS transistors within the current mirror, which is the dynamic element-matching technique [24].

### 2.2. V-Curve Compensation Technique

**Figure 5:**
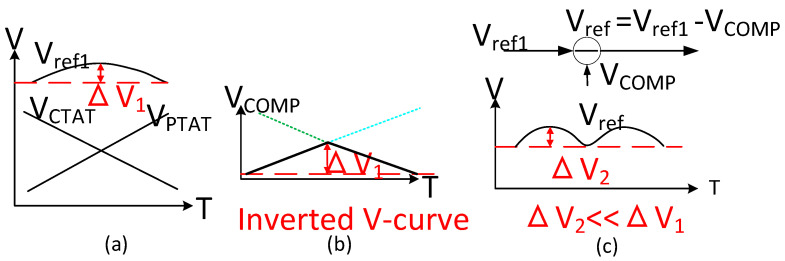
V-curve compensation principle. (**a**) Generation of first order voltage reference; (**b**) Principle of the inverted V-curve; (**c**) Ideal curve of the proposed bandgap reference.

**Figure 6:**
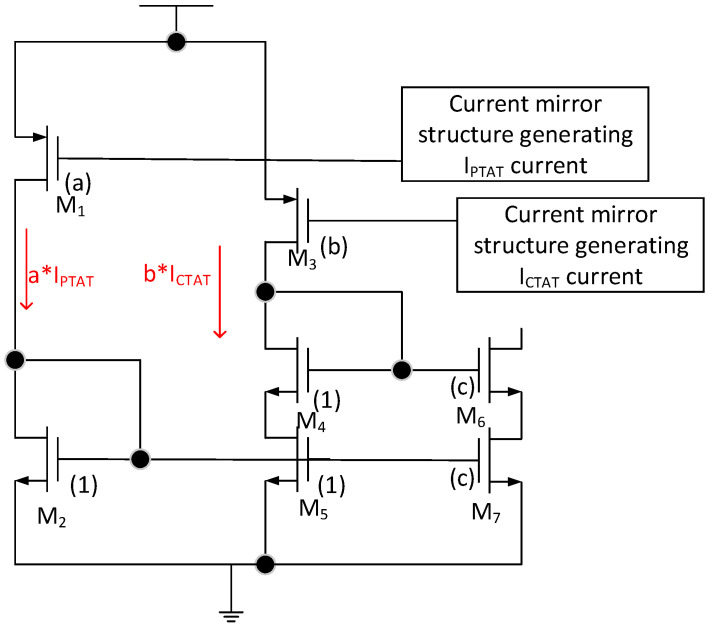
V-curve compensation structure.

To obtain the ultra-low temperature coefficient, the inverted V-curve structure is employed, which can further compensate the zero-TC (temperature coefficient) current. The principle of V-curve compensation is shown in Figure 5. Figure 5a shows the generation of traditional first-order zero-TC bandgap reference. V_ref1_ with the voltage variation $∆V_{1}$ is generated by the combination of V_CTAT_ and V_PTAT_. Figure 5b shows the principle of the creation of the inverted V structure with the voltage variation $∆V_{1}$. Finally, the ideal temperature curve of the proposed bandgap reference, which exhibits a voltage variation $∆V_{2}$, is shown in Figure 5c. It can be seen that $∆V_{2}$ is far less than $∆V_{1}$. The core circuit of the compensation is shown in Figure 6; I_PTAT_ and I_CTAT_ are mirrored with weight a/b by the transistors M_1_ or M_3_. When the current a·I_PTAT_ is smaller than b·I_CTAT_, the transistor M_3_ is in the linear region and a·I_PTAT_ becomes the dominant current through M_3_, M_4_, and M_5_. When the current a·I_PTAT_ is larger than b·I_CTAT_, the region of the transistor M_5_ will be changed into the triode region, and b·I_CTAT_ becomes a dominant current. In addition, the current flowing through M_6_ and M_7_ is mirrored from the current through M_4_ and M_5_ with a weight of c. As shown in Equation (5), the compensation current I_comp_ is equal to a·c·I_PTAT_ when a·I_PTAT_ becomes the dominant current; otherwise, I_comp_ is equal to b·c·I_CTAT_.

(5) $$I_{\mathrm{COMP}}=\{\begin{array}{cc} a\cdot c\cdot I_{\mathrm{PTAT}} & (a\cdot I_{\mathrm{PTAT}}<b\cdot I_{\mathrm{CTAT}\;})\\ b\cdot c\cdot I_{\mathrm{CTAT}\;} & (a\cdot I_{\mathrm{PTAT}}>b\cdot I_{\mathrm{CTAT}\;}) \end{array}$$

**Figure 7:**
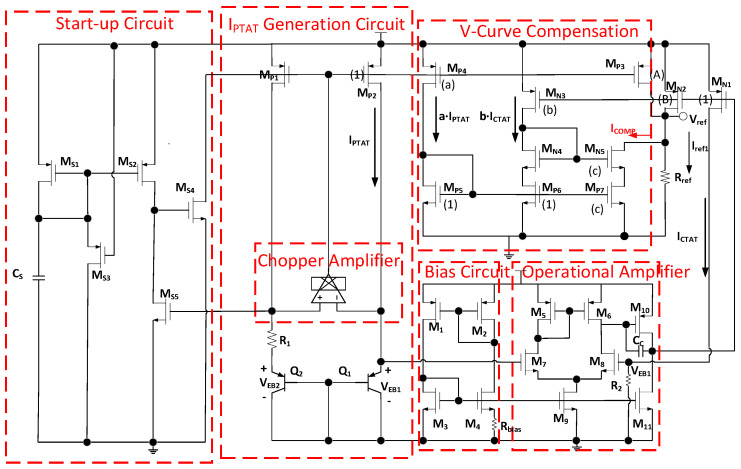
Overall bandgap reference circuit.

The overall circuit structure is shown in Figure 7. The bandgap reference comprises start-up circuit, I_PTAT_ generation circuit with chopper-operational amplifier, a bias circuit, operational amplifier, and V-curve structure. The principle of the start-up circuit is as follows. When the circuit is powered on, the C_S_ begin to charge. The process of charging the capacitor is from a low level to high level, meaning that it starts with a low level during charging, causing M_S4_ to conduct, which pulls down the gate voltages of M_P1_ and M_P2_, eventually turning them on. As M_P1_ conducts, a current is generated, flowing through the BJTs and the resistor R_1_ to form a voltage of about 780 mV, thus turning on M_S5_. As M_S5_ conducts, M_S4_ turns off. Simultaneously, as the capacitor C_S_ charges, the gate voltages of M_S1_ and M_S2_ continue to rise and eventually turn off. Ultimately, M_S5_ operates in the linear region, with the other transistors being in the cutoff region. During power-down, M_S3_ conducts, allowing the capacitor C_S_ to discharge and return to its initial state, ready for the next power-up. The bias current, which remains constant regardless of the power supply voltage, is provided by the bias circuit. Therefore, the proposed bandgap reference can achieve high PSRR. The operational amplifier is composed of two amplifier stages, which include a five-transistor OTA (Operational Transconductance Amplifier) and a common-source amplifier. Additionally, it utilizes Capacitor C_C_ for Miller compensation to enhance the phase margin of the system, which increases the stability of the operational amplifier. The operational amplifier is biased in the deep negative feedback in order to ensure the gate voltages of M_7_ and M_8_ to be equal. The area ratio of Q_2_ and Q_1_ is 8:1, which can achieve optimal possible layout matching. When Q_1_ and Q_2_ operate at different current densities, the difference(ΔV_EB_) in the two junction voltages (V_EB1_ and V_EB2_) has a positive TC of approximately 0.194 mV/°C (4 μA operating current). While the emitter-base junction voltage V_EB1_ of Q_1_ has a negative TC of about −1.575 mV/°C. Through R_2_, the negative-TC current I_CTAT_ is generated, and its value is V_EB1_/R_2_. The current mirror structure composed of M_N1_ and M_N2_ duplicates the I_CTAT_ current with Weight B. When the I_CTAT_ is combined with the I_PTAT_, which is ΔV_EB_/R_1_ with Weight A, the expression of I_ref1_ is shown in Equation (6). Without the V-curve compensation circuit, the I_ref1_ directly flows to the R_REF_ resistor and achieves a first-order bandgap reference. In addition, with the V-curve compensation structure, the current of R_ref_ is equal to I_ref1_ minus I_COMP_, as the I_COMP_ flows into the V-curve compensation structure, realizing the second-order compensation. The proposed bandgap reference voltage is provided by the Equation (7).

(6) $$I_{\mathrm{ref}1}=A\cdot I_{\mathrm{PTAT}}+B\cdot I_{\mathrm{CTAT}}=A\cdot \frac{{∆V}_{\mathrm{EB}}}{R_{1}}+B\cdot \frac{V_{\mathrm{EB}1}}{R2}$$

(7) $$\mathrm{Vref}\;={V_{ref1}-V_{COMP}=(I}_{ref1}\;-\;I_{COMP\;})\cdot R_{ref}$$

## 3. Results

**Figure 8:**
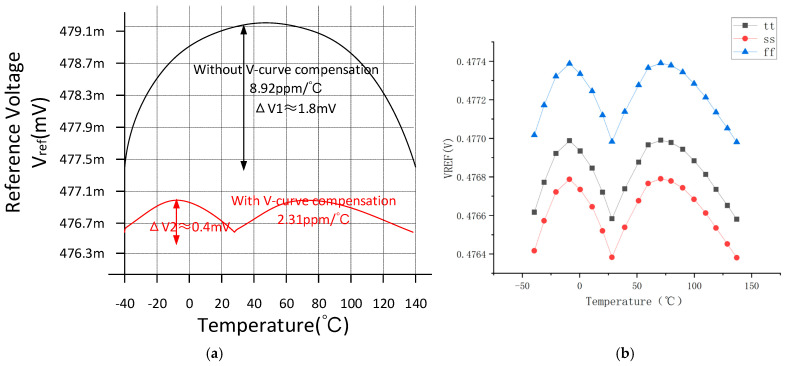
(**a**) Temperature-curve simulation result plot; (**b**) Temperature-curve simulation at different corners.

The proposed circuit was integrated using a standard 0.18 μm CMOS technology at 1.8 V supply. Figure 8a shows the simulation results of an uncompensated and compensated bandgap reference across a temperature range of 180 °C (−40–140 °C). Compared with the simulation results of a traditional bandgap reference, the proposed bandgap reference shows a significant 74.1% decrease in TC, decreasing from 8.92 to 2.31 ppm/°C. The variation in the reference voltage has been reduced from 1.8 mV to 0.4 mV. Figure 8b shows the variation of V_ref_ with the temperature for different corners over a temperature range of −40 °C to 140 °C. The proposed bandgap reference V_ref_ maximum value is 0.4774 V, and the minimum value is 0.4763 V. In addition, the TCs are 2.31, 2.34, and 2.79 ppm/°C for TT, SS, and FF corners, respectively.

**Figure 9:**
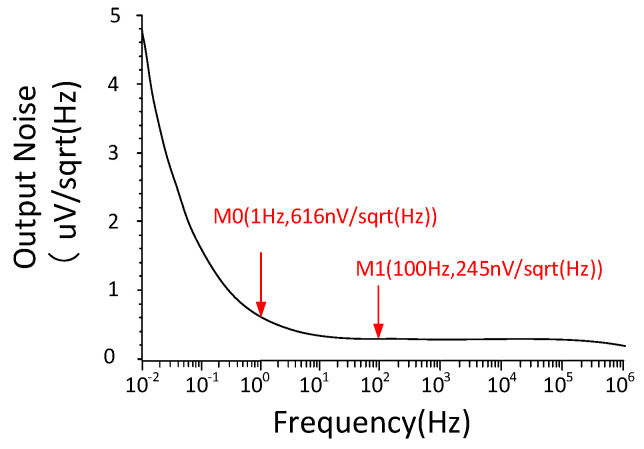
Output noise-simulation plot.

Figure 9 presents the results of the Noise Spectrum simulation (e.g., PSS + Pnoise simulation using Cadence tools) at the output of the bandgap reference. The output noise density is decreased with the frequency, from 4.8 μV/√Hz to 190 nV/√Hz, over a frequency range of 0.01–1 MHz. In the MEMS sensor-interface circuit, the most critical aspect of concern is the low frequency, ranging from 1 to 100 Hz. By examining Points M_0_ and M_1_, it is found that the output voltage noise density of the proposed bandgap reference voltage source is 616 nV/√Hz at 1 Hz, and 245 nV/√Hz at 100 Hz. These results demonstrate that the proposed bandgap reference can satisfy the low-frequency noise requirements of the MEMS sensor-interface circuits.

**Figure 10:**
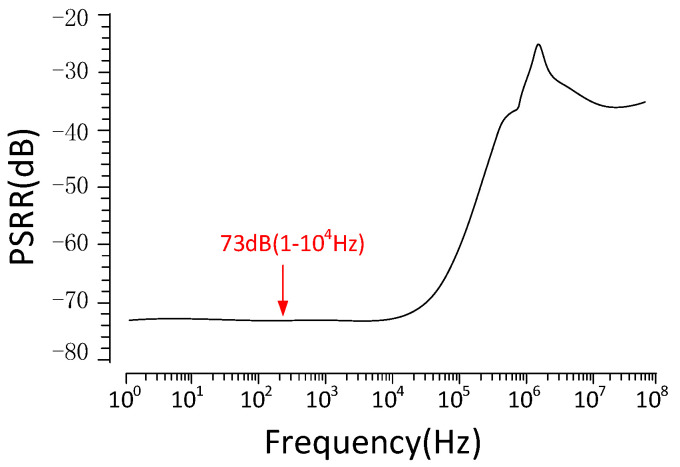
PSRR of the Vref simulation results of the proposed bandgap reference.

**Figure 11:**
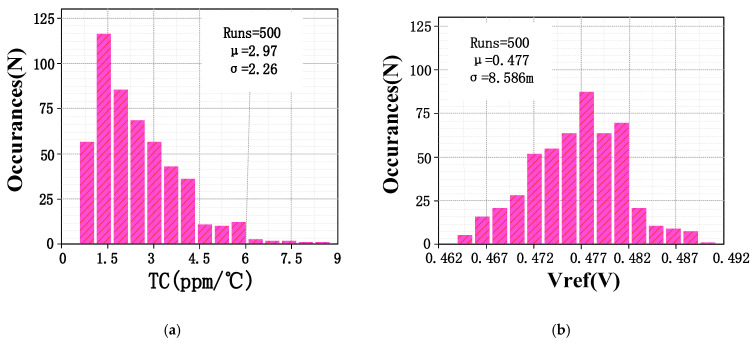
(**a**) Monte Carlo simulation results of the TC; (**b**) Monte Carlo simulation result of Vref.

**Figure 12:**
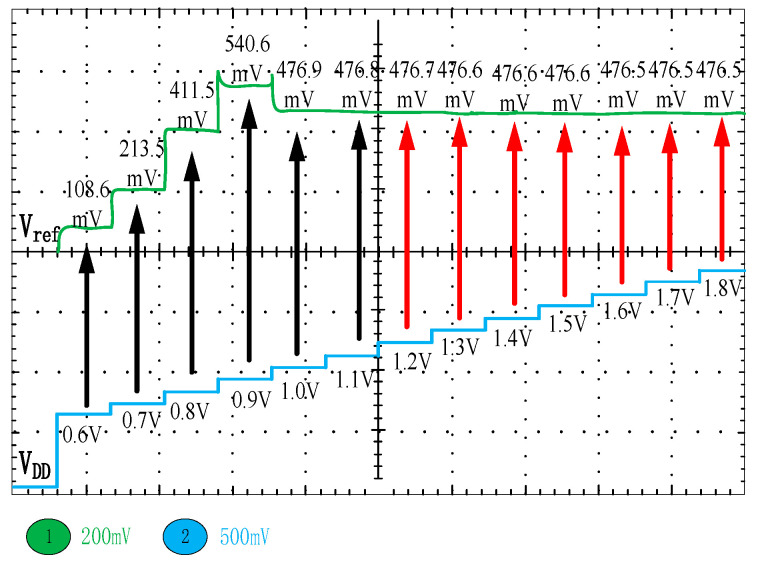
Measured output voltage at each step voltage from 0.6 to 1.8 V.( The black arrow represents the portion within the measurement range that does not belong to the linear regulation rate measurement range, while the red portion represents the linear regulation rate measurement range).

Figure 10 shows the PSRR of the V_ref_ simulation result. The proposed bandgap reference achieves PSRR as high as 73 dB at low frequencies(<10 kHz). The PSRR decreases at frequencies above 10 kHz. Figure 11a,b show the results of 500 Monte Carlo runs for TC and Vref, respectively. The average TC of the proposed circuit is 2.97 ppm/°C, and the standard deviation is 2.26 ppm/°C. In addition, the average output voltage V_ref_ is 477 mV with a standard variation of 8.586 mV. Figure 12 illustrates the V_ref_ values corresponding to each step voltage, which were obtained through the application of a staircase sweep voltage from 0.6 to 1.8 V under room-temperature conditions (27 °C) (measurement was conducted using Tektronix’s MSO 2-series oscilloscope). The experimental result demonstrates a steady output voltage of 476.5 mV, exhibiting minimal variation of just 0.2 mV in the presence of a 0.6 V supply-voltage fluctuation. This implies that the line regulation of the proposed bandgap reference is 0.33 mV/V within a voltage supply range of 1.2 V to 1.8 V. The power consumption of the bandgap reference presented in this paper is 107 μW.

**Figure 13:**
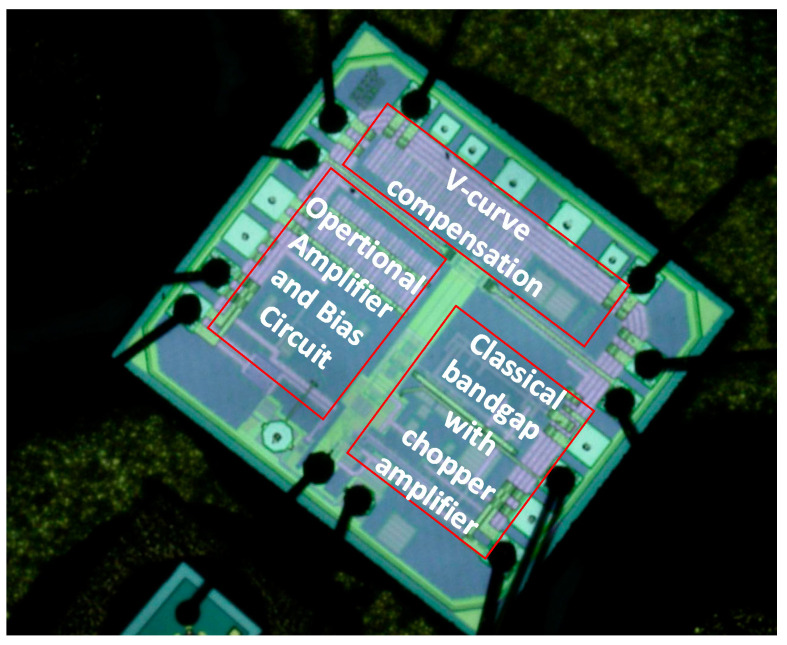
Photo of the bandgap reference chip.

Photo of the bandgap reference chip is shown in Figure 13, and the active area is 0.21 × 0.28 mm^2^. Table 1 provides a performance comparison between this study and other relevant research. Through comparison, the improvements of the proposed circuit in terms of noise and TC are significant, and the performance of PSRR is also commendable. Overall, the proposed bandgap reference is suitable for the MEMS sensor-interface circuit.

## 4. Discussion

A low-noise, high-precision bandgap reference is proposed in this paper, and the innovations of the chopper-operational amplifier of the proposed circuit are as follows. Firstly, instead of using choppers directly at the output, choppers are used at the low-impedance nodes of the folded cascode structure. As analyzed previously, placing chopper at low-impedance nodes minimally affects the amplifier’s bandwidth while allowing for a larger output swing. Secondly, the method of chopping at low-impedance nodes means that the modulation switches are not at the direct output end, so the switching noise is not directly coupled to the output, thus avoiding significant switching noise interference and allowing for a higher chopping-signal frequency. Additionally, Chopper CH_3_ is added in the middle of the low-voltage cascode current mirror, using dynamic element-matching techniques to further reduce current mismatch and input offsets in the current mirror. Furthermore, a virtual switch structure is employed to further reduce charge injection and clock feedthrough.

However, there are also some limitations in the proposed circuit. The proposed bandgap reference is primarily employed in sensor interface circuits for low-frequency signal acquisition. Hence, it has certain frequency limitations. Its ability to suppress high-frequency noise is relatively limited. Moreover, the three chopping switches increase the circuit’s power consumption and introduce a certain level of residual offset voltage. Additionally, the proposed circuit needs additional structure to produce accurate two-phase, non-overlapping clock signal, which will increase the difficulty of design. It also introduces a mirror pole, as shown in Point X in Figure 4. This will impact the speed of the feedback system in applications utilizing this structure.

In general, the proposed bandgap reference can be applied to MEMS sensor-interface circuits, such as MEMS-tunneling magnetoresistance sensor-interface circuits, MEMS accelerometer sensor-interface circuits, and MEMS gas-sensor-interface circuits. MEMS sensor-interface circuits are indispensable for micromachines because they help achieve reliable data acquisition, signal processing, and communication, thereby enhancing the performance and application scope of micro-mechanical systems. These interface circuits ensure effective collaboration between sensors and other systems, enabling a wide range of measurement, control, and feedback applications.

## 5. Conclusions

A low-noise and low-temperature coefficient bandgap reference based on 0.18μm CMOS technology is proposed in this paper. By employing a chopper operational amplifier, the output low-frequency noise and offset of the bandgap reference is significantly reduced. In addition, the V-curve compensation technique is utilized to further compensate for the temperature coefficient of the bandgap reference. Results indicate that the high precision of the proposed bandgap reference enables it to meet the requirements of MEMS sensor circuits and makes it well-suited for other high-precision applications.

## Figures and Tables

**Table 1:** Performance comparison.

	Design	[15]	[16]	[18]	[21]	[This Work]
Parameter						
Process (μm)		0.18COMS	0.065CMOS	0.18BCD	0.15CMOS	0.18CMOS
Output noise (nV/$\sqrt{\mathrm{Hz}}$)		1420 @320 Hz	880 @10Hz	442 @0.1–10 Hz	——	616 @1 Hz
Temperature coefficient (ppm/°C)		5–15	13.03	13.07	1.03	2.31
Power supply (V)		2.7–3.3	——	5	1.8	1.8
Power supply-rejection ratio (dB)		80@DC	80@1 MHz	81.28@10 Hz	83@100 Hz	73@10 kHz
Line regulation (mV/V)		0.05	——	——	0.2	0.33

## Data Availability

The data presented in this study are available on request from the corresponding author.
